# High-Density Lipoproteins from Coronary Artery Disease and Aortic Valve Stenosis Patients Differentially Regulate Gene Expression in a Model of Cardiac Adipocytes

**DOI:** 10.3390/cells14030205

**Published:** 2025-01-30

**Authors:** María Luna-Luna, Araceli Páez, Felipe Massó, Rebeca López-Marure, Jorge Moisés Zozaya-García, Ariana Vargas-Castillo, Daniel Gómez-Pineda, Armando R. Tovar, Jonathan J. Magaña, José Manuel Fragoso, Margarita Gutiérrez-Saldaña, Zuriel Téllez-Osorio, Óscar Pérez-Méndez

**Affiliations:** 1 Department of Molecular Biology, Instituto Nacional de Cardiología “Ignacio Chávez”, Mexico City 14080, Mexico; luisdaniel.gp.28@gmail.com (D.G.-P.); mfragoso1275@yahoo.com.mx (J.M.F.); 2182033925@alumnos.xoc.uam.mx (M.G.-S.); nztm00@gmail.com (Z.T.-O.); 2 Unidad de Investigación UNAM-INCICH, Instituto Nacional de Cardiología Ignacio Chávez and Instituto de Investigaciones Biomédicas, Universidad Nacional Autónoma de México, Mexico City 04510, Mexico; arapaez@yahoo.com.mx (A.P.); f_masso@yahoo.com (F.M.); 3 Department of Physiology, Instituto Nacional de Cardiología “Ignacio Chávez”, Mexico City 14080, Mexico; rlmarure@yahoo.com.mx; 4 Department of General and Endoscopic Surgery, Hepatic and Bile Ducts Clinic, Hospital General “Dr. Manuel Gea González”, Mexico City 14080, Mexico; jmoisesz@hotmail.com; 5 Department of Cancer Biology, Dana-Farber Cancer Institute, Boston, MA 02215, USA; arianae_vargas-castillo@dfci.harvard.edu; 6 Department of Cell Biology, Harvard Medical School, Boston, MA 02115, USA; 7 Nutrition Physiology Department, Instituto Nacional de Ciencias Médicas y Nutrición Salvador Zubirán, Mexico City 14080, Mexico; tovar.ar@gmail.com; 8 Laboratory of Genomic Medicine, Department of Genetics, National Rehabilitation Institute Luis Guillermo Ibarra Ibarra (INRLGII), Mexico City 14389, Mexico; magana.jj@tec.mx; 9 Tecnologico de Monterrey, Engineering School, Campus Ciudad de Mexico, Mexico City 14380, Mexico

**Keywords:** osteopontin, bone morphogenetic protein-2, bone morphogenetic protein-4, leptin, uncoupling protein-1, perilipin-2, adipose tissue, high-density lipoproteins

## Abstract

Previous reports have described a statistical association between high-density lipoproteins (HDL) subclasses and the expression of genes coding for pro-calcifying proteins in the epicardial adipose tissue of patients with coronary artery disease (CAD) and aortic valvular stenosis (AVS). These results suggest a causal relationship between HDL and the regulation of gene expression in epicardial adipose tissue. However, there is no experimental evidence that supports this causal relationship. Therefore, we explored the effect of HDL isolated from CAD or AVS patients on the expression of *OPN*, *BMP2*, and *BMP4*, genes coding for proteins related to calcification, osteopontin, and bone morphogenetic proteins -2 and -4, respectively, and *LEP*, *UCP,* and *PER*, coding for leptin, uncoupling protein-1, and perilipin-2, respectively, proteins that confer phenotypic characteristics to adipocytes. The experiments were performed using a novel model of cardiac adipocytes differentiated in vitro from stromal cells of rabbit cardiac adipose tissue. AVS or CAD patients’ HDL differentially modulated the expression of *BMP4* and *LEP*, whereas HDL from both kinds of patients upregulated the *OPN* gene expression. A high concentration of triglycerides associated to small HDL and a higher concentration of phospholipids of large HDL from CAD patients than those from AVS individuals were the most remarkable structural differences. Finally, we demonstrated that cholesterol from reconstituted HDL was internalized to the adipocytes. The regulation of genes related to the secretory activity of cardiac adipocytes mediated by HDL has clinical implications as a potential therapeutic target for the prevention and treatment of CAD and AVS. In summary, the HDL isolated from the CAD and AVS patients differentially regulated gene expression in adipocytes by a mechanism that seems to be dependent on HDL lipid internalization to the cells and structural characteristics of the lipoproteins.

## 1. Introduction

High-density lipoproteins (HDL) are macromolecular aggregates composed of cholesterol and phospholipids on the surface and triglycerides and esterified cholesterol in the core [1,2]. Apolipoprotein (apo) A-I is the main protein that stabilizes these aggregates [3,4], but more than 100 proteins have been described as part of the HDL structure [3,5]. These lipoproteins are considered anti-atherogenic particles because of the inverse correlation between HDL cholesterol (HDL-C) plasma concentrations and the incidence of coronary artery disease (CAD) risk [6,7]. This cardio-protector role of HDL has been explained by reverse cholesterol transport (RCT) [8], where these lipoproteins play a central role. However, drugs based on RCT, such as CETP inhibitors, failed to reduce the risk of CAD [9,10], stressing this intravascular pathway’s validity as the potential explanation for the anti-atherogenic role of HDL. In addition, carriers of apo A-I mutations, whose HDL-C plasma concentrations are very low, do not show an increased risk of CAD [9]. In the same vein, about half of the patients with CAD clinical events have normal levels of HDL-C [9,11,12,13]. These pieces of evidence indicate that the HDL physiological function is beyond their cholesterol content and establishes the necessity of new paradigms that explain the cardio-protective role of HDL [9].

In this context, it was demonstrated that HDL isolated from the plasma of CAD or aortic valve stenosis (AVS) patients differed in lipid composition and relative subclass proportion [14]. Moreover, both characteristics were statistically associated with the level of expression of genes related to coronary calcification in epicardial adipose tissue (EAT) [14], which is an atheroma progression marker. These results suggest that the cardio-protector role of HDL may be related to the capacity to regulate DNA transcription in epicardial adipose tissue; such HDL activity results are attractive in explaining some properties of these lipoproteins. Previous results from our laboratory showed that one mechanism by which HDL modifies cell physiology is by delivering functional lipids to cells [15]. Although gene regulation by HDL has been reported in some cell types, including macrophages [16,17], little is known about whether HDL can regulate gene expression in cardiac adipocytes, thus determining intracellular mRNA levels, and whether such a property may depend on HDL structure and HDL internalization to the cells. Therefore, the aim of this study was to explore the effect of lipid composition and size distribution of HDL isolated from CAD or AVS patients on the expression of the *OPN*, *BMP2*, *BMP4*, *LEP*, *UCP*, and *PER* genes in a new model of cardiac adipocytes. *OPN*, *BMP2*, and *BMP4* code for osteopontin and bone morphogenetic proteins -2 and -4, respectively, proteins related to calcium deposition in tissues [18], and the *LEP*, *UCP,* and *PER* genes code for proteins that confer phenotypic characteristics to adipocytes, i.e., leptin, uncoupling protein-1, and perilipin-2, respectively [19].

## 2. Materials and Methods

### 2.1. Patients

Eleven patients with a diagnosis of coronary artery disease (CAD) and ten patients with aortic valve stenosis and without CAD who were candidates for revascularization surgery or chirurgical valve replacement, respectively, were enrolled in the study. Patients with thyroid, renal, or hepatic diseases, with previous coronary angioplasty or previous revascularization surgery, and patients without informed consentient signed were excluded.

This study was approved by the Ethics Committee of the National Institute of Cardiology “Ignacio Chávez” with the number 13-818.

### 2.2. Laboratory Assessment

Blood samples from patients were obtained in EDTA tubes after 12 h of overnight fasting. The samples were centrifugated at 4 °C to separate the plasma and frozen at −20 °C until use.

Enzymatic colorimetric methods (Randox LTD, Crumlin, UK) were used to determine plasma cholesterol (C), triglycerides (TG), and glucose concentrations. HDL lipids were determined by previous precipitation of the apo B-containing lipoproteins by the phosphotungstic acid-Mg^2+^ method. Low-density lipoprotein -cholesterol (LDL-C) was estimated with the formula of Friedewald.

### 2.3. Isolation of HDL

Apo B-containing lipoproteins were eliminated by sequential ultracentrifugation by adjusting the plasma density to 1.063 g/mL with solid KBr as previously described [20]. The conditions of separation were 100,000 rpm, 2.5, hours, and 10 °C. Infranatant density was increased to 1.21 g/mL with KBr, and the samples were centrifuged for 3 h at 100,000 rpm and 10 °C. The supernatant containing the HDL was dialyzed against phosphate buffer (150 mM NaCl, 10 mM Na_2_HPO_4_/NaH_2_PO_4_), pH 7.4.

### 2.4. Analysis of HDL Subclasses

HDL were separated by non-denaturing 3–30% polyacrylamide gradient gel electrophoresis at 180 V for 22 h [20]. Semi-liquid enzymatic mixtures to stain cholesterol, triglycerides, and phospholipids were elaborated as previously described [20]. Briefly, the gels were incubated at 37 °C for 30 min in darkness with the enzymatic mixtures, washed with water, and scanned (lipid image). The gels were then distained using a 25% methanol–10% acetic acid–65% water solution, and re-stained with Coomassie blue R-250 and scanned again (protein image). The images were analyzed using VisionWorks 8.20 version (Ultra-Violet Products Ltd., Nuffield, Cambridge, UK), and the area of the lipid or protein of each subclass was expressed as a percentage. The size intervals of reference to each subclass used were HDL3c, 7.94–8.45 nm; HDL3b, 8.45–8.98 nm; HDL3a, 8.98–9.94 nm; HDL2a, 9.94–10.58 nm; and HDL2b, 10.58–13.59 nm [20].

The lipid concentration of the HDL subclasses was calculated on the basis of the lipid percentage of each subclass and its corresponding HDL-lipid plasma concentration.

### 2.5. Cell Culture

Heart adipose tissue fragments of about 1 mm^3^ from male New Zealand White rabbit were digested with collagenase type I (Gibco, Life Technologies, Carlsbad, CA, USA) at 0.05% and at 37 °C with gentle stirring. The mixture was centrifugated at 600× *g* for 10 min and the supernatant was eliminated. The pellet, which contained the stromal vascular fraction cells (SVFC), was aliquoted and frozen until use.

For cell differentiation, the SVFC were resuspended in DMEM/F12 medium containing phenol red and supplemented with 10% fetal bovine serum (FBS) and 100 U/mL of a penicillin–streptomycin mixture. Stromal cells were cultured and expanded in flasks of 75 cm^2^ at 37 °C with 5% pCO_2_ in a humidified atmosphere until a confluence of 85%. Stromal cells were cultured at a cell density of 100,000 cells/well and incubated overnight at 37 °C with 5% pCO_2_ in a humidified atmosphere until they reached confluence. This primary culture was reseeded up to 4 times. For this study, all experiments were performed with the same stromal cells.

### 2.6. Differentiation from Stromal Cells to Adipocytes

Based on the adipocyte differentiation induction of 3T3-L1 cells [21,22], we used two types of media as follows: (1) differentiation medium, which contains DMEM/F12 medium supplemented with 10% FBS, 100 U/mL penicillin–streptomycin mixture, 0.5 mM 1-methyl-3-isobutylxantine (IBMX), 5 μM dexamethasone, 200 μM indomethacin, and 10 μg/mL insulin, and (2) maintenance medium, whose composition was DMEM/F12 medium supplemented with 10% FBS, 100 U/mL penicillin–streptomycin mixture, and 10 μM insulin.

The cells were stimulated with a differentiation medium for 4 days, and then it was replaced with a maintenance medium for 2 days. This scheme was repeated for 3 weeks. The conditions of incubation were 37 °C and 5% pCO_2_. During the differentiation process, cultures that lost confluence, below 70%, were discarded. The homogeneity of the batches of differentiated cells was warranted by the perilipin mRNA levels.

### 2.7. Oil Red Stain

An oil red stain was performed to confirm the formation of the lipid drops in the cells, differentiated adipocytes. Briefly, cells were fixed with formalin 4%, washed with distilled water, and stained with fresh 0.5% Oil Red O solution for 15 min. Cells were dipped in 60% isopropyl alcohol and washed with distilled water.

### 2.8. Stimulation of Differentiated Adipocytes with HDL

Differentiated adipocytes were incubated for 1 h with DMEM/F12 medium supplemented with 7% lipoprotein-poor FBS. Then, HDL were slowly added at a final cholesterol concentration of 25 mg/dL and incubated for 8 h at 37 °C and 5% pCO_2_. Medium-free cells were harvested with Trizol (Amresco, OH, USA) and stored at −70 °C until use. All experiments were performed in duplicate.

### 2.9. Gene Expression Assay

Total RNA was isolated from differentiated adipocytes using the RiboPure™ RNA Purification Kit following the manufacturer’s instructions (Ambion, Life Technologies, Carlsbad, CA, USA). RNAse-free DNAse I (Invitrogen, Life Technologies, Carlsbad, CA, USA) was used to eliminate the contaminant DNA; the enzyme was inactivated with the addition of 2.5 mM EDTA final concentration and incubated for 10 min at 75 °C. The RNA quantity, purity, and integrity were determined by spectrophotometry and agarose gels, respectively.

RNA was reverse transcribed using SuperScript^®^ VILO™ cDNA Synthesis Kit (Invitrogen, Life Technologies, Carlsbad, CA, USA) in line with the manufacturer’s specifications.

Gene expression of *OPN*, *BMP2*, *BMP4*, *LEP*, *UCP1*, and *PER2* was performed by qPCR on a 7900HT Fast Real-Time PCR system (Applied Biosystems, Foster City, USA) using 50 ng cDNA, Maxima SYBR Green/ROX qPCR Master Mix (2×) (Thermo Scientific, Thermo Fisher Scientific Baltics UAB, Vilnius, Lithuania), and *GAPDH* as a housekeeping gene. The primers used are shown in Supplementary Table S1, and the cycle conditions were as follows: initial denaturation at 95 °C for 10 min followed by 40 amplification cycles with denaturing (95 °C for 30 s), primer annealing (Tm for 30 s), and extension (72 °C for 40 s). Gene expression was calculated by the standard curve method and normalized to *GAPDH*.

### 2.10. Synthesis of Reconstituted HDL (rHDL)

rHDL were prepared with phosphatidylcholine and either, only free cholesterol, or a free cholesterol and fluorescent cholesterol (25-nitrobenzoxadiazole (NBD)-cholesterol) mixture and dissolved in chloroform at mass proportions of 95%, 5%, or 2.5%/2.5%, respectively. The lipids were mixed in a glass conic vial and vigorously vortexed, as previously described [15]. The mixture was dried under agitation with a nitrogen stream to form the film of lipids. Posteriorly, 0.5 mg human Apo AI and 10 mM Tris–140 mM NaCl–1 mM EDTA buffer were added to the film, followed by sodium cholate (final concentration 310 mg/mL), under vigorous stirring. The mixture was dialyzed with 10 mM PBS for three days and was filtered through a 0.22 μM syringe filter.

rHDL without NBD cholesterol were labeled in the protein moiety with Alexa Fluor 568 Molecular Probes according to the manufacturer’s instructions (Life Technologies, Eugene, OR, USA).

### 2.11. Internalization Assay of HDL

Differentiated adipocytes were incubated with DMEM/F12 supplemented with poor-lipid FBS at 7%, and they were incubated at 37° and 5% pCO_2_ for 1 h. The medium was replaced with a new medium supplemented with rHDL labeled with NBD or Alexa Fluor 568 at a final concentration of 100 mg of protein; the cells were incubated at 37° and 5% pCO_2_ for two hours.

The medium was removed, and the cells were washed twice with 10 mM PBS. Cells were fixed with paraformaldehyde at 4% and incubated for 15 min in the dark at 4 °C. Then, the plate was washed with PBS and the nuclei were stained with 4′,6-diamidino-2-phenylindole (DAPI). Images were obtained using an LSM-700 Carl Zeiss confocal microscope (Carl Zeiss, Baden-Württemberg, Germany).

### 2.12. Statistical Analysis

Data are shown as median and interquartile range or as percentages. Data analysis was performed with Graph Pad Prisma software 5.0 (La Jolla, CA, EE.UU.). Kruskal–Wallis and Dunn’s tests were used to perform multiple comparisons. The comparisons between two groups were performed using the Mann–Whitney *U*-test. A *p* < 0.05 was considered as statistically significant.

## 3. Results

### 3.1. Patients’ Clinical Characteristics

We enrolled 10 patients with aortic valve stenosis (AVS) without coronary calcification determined by computed tomography and 11 patients with clinical manifestations of CAD and coronary calcium score > 1 (Table 1). Most parameters were statistically similar except for the systolic blood pressure, which was higher (11%) in the CAD group compared to AVS patients (Table 1).

### 3.2. Differentiated Adipocytes

**Figure 1:**
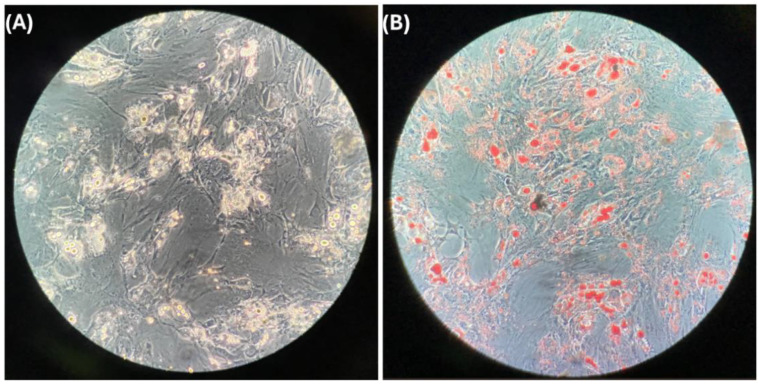
Differentiated adipocytes in vitro: (**A**) characteristic image of preadipocytes differentiated to adipocytes in vitro and (**B**) stain of lipid drop of adipocytes with oil red.

**Figure 2:**
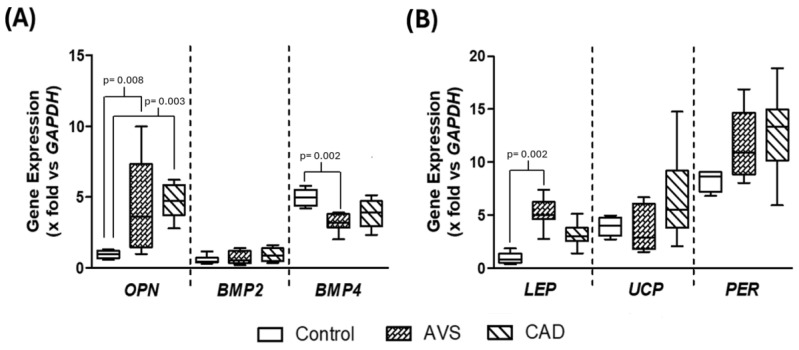
Levels of mRNA of (**A**) genes related to calcification and (**B**) characteristic genes of adipose tissue. Primary cultures of in vitro differentiated adipocytes were incubated in the absence of HDL (control) or stimulated for 8 h with HDL isolated from patients with CAD or AVS. Relative mRNA expression was quantified by qPCR and normalized by the *GAPDH* gene expression. AVS: aortic valve stenosis; CAD: coronary artery disease; *OPN*: osteopontin; *BMP2*: bone morphogenetic protein-2; *BMP4*: bone morphogenetic protein-4; *LEP*: leptin; *UCP*: uncoupling protein-1; *PER*: perilipin-2; *GAPDH*: glyceraldehyde-3-phosphate dehydrogenase. Data are expressed as median and interquartile range. Groups were compared by a Kruskal–Wallis test; individual *p*-values were calculated by a Mann–Whitney *U*-test.

We have previously demonstrated a statistical relationship between the expression of genes related to the calcification of atheroma in epicardial adipose tissue and the subclasses of HDL [14]. To determine whether this statistical association may be due to a biological effect, we used in vitro differentiated adipocytes from rabbit cardiac adipose tissue (Figure 1A). Notably, rabbits share important similitudes with humans in their lipoprotein metabolism [23]. Differentiated adipocytes were characterized by an important accumulation of lipids in their cytoplasm (Figure 1B) and the concomitant expression of genes coding for leptin (*LEP*) uncoupling protein-1 *(UCP*) and perilipin-2 (*PER*), confirming the phenotype of adipocytes (Figure 2B). All cultures were derived from the same rabbit cardiac adipose tissue; in addition, the perilipin relative expression was within a narrow interval, 8.7 [7.2–9.1], indicating homogeneity of the cell cultures throughout the study.

### 3.3. Gene Expression in Differentiated Adipocytes Stimulated with HDL

Differentiated adipocytes were stimulated with HDL isolated from AVS and CAD patients at a final cholesterol concentration of 25 mg/dL. Under these conditions, the incubation of adipocytes in the presence of HDL isolated from any AVS or CAD patients clearly increased the *OPN* gene expression compared to control cells incubated in the absence of HDL (Figure 2A). Another pro-calcifying protein gene modified by HDL was *BMP4*; significantly lower mRNA levels were observed when adipocytes were incubated with HDL isolated from AVS patients, whereas the HDL from CAD patients did not affect *BMP4* gene expression compared to control cells (Figure 2A).

Concerning the characteristic genes of adipocytes, the presence of HDL from AVS patients in the culture media was associated with increased mRNA levels of *LEP*. A similar tendency was observed with HDL isolated from CAD patients, but the difference did not reach statistical significance (Figure 2B). *UCP* and *PER* gene expressions were not affected by HDL. Importantly, there were no differences in the mRNA levels between the cells stimulated with HDL isolated from CAD and AVS patients.

### 3.4. Internalization of HDL Cholesterol

**Figure 3:**
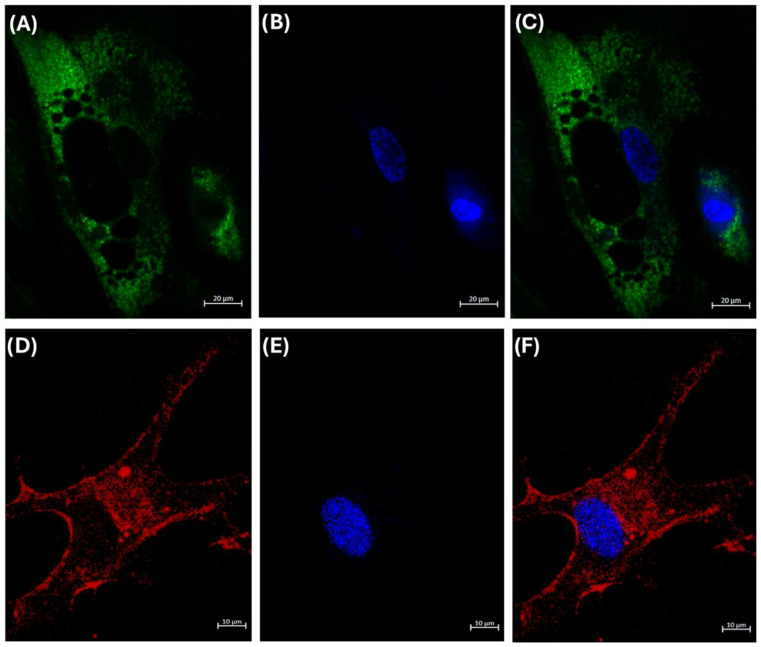
Representative confocal image of internalization of cholesterol (**A**–**C**) and protein (**D**–**F**) of HDL into adipocytes. Cultured adipocytes were incubated with rHDL that contained fluorescent 25-nitrobenzoxadiazole (NBD)-cholesterol (green, (**A**–**C**)) or protein labeled with Alexa Fluor 568 (red, (**D**–**F**)). After incubation, cells were fixed, and nuclei were stained with the fluorophore 4′,6-diamidino-2-phenylindole (DAPI) (blue). Confocal microscope images of the cholesterol (**A**) and protein (**D**) of rHDL in the cytoplasm of the cells. (B and E) Confocal microscope images of the nuclei of the cells. (**C**) Merge of images (**A**) and (**B**); (**F**) merge of images (**D**) and (**E**). The dark zones inside the cytoplasm correspond to the unstained lipid drops of the adipocytes. The scale bar is indicated in all the confocal microscope images.

As stated above, a mechanism by which HDL could regulate gene expression is by delivering cholesterol to cells, thereby modifying the fluidity of their membranes [15,24]. However, this property has not been demonstrated in adipocytes. Therefore, to explore whether adipocytes can internalize HDL into their cytoplasm, we incubated our model of adipocytes with rHDL, whose cholesterol was labeled with NBD [15]. We observed that the adipocytes internalized the cholesterol of HDL, and it was distributed in the whole cell except for the lipid drops (Figure 3).

### 3.5. Composition and Relative Proportion of HDL Subclasses

HDL-C internalization supports the hypothesis that HDL contain functional lipids that can regulate gene expression. In this way, the detailed characterization of HDL subclasses of patients became imperative. Therefore, although we did not find statistical differences in the plasma concentrations of HDL-lipids (Table 1), we determined the lipid concentrations in each subclass of HDL isolated by gradient gel electrophoresis and enzymatically stained [20].

The cholesterol concentration of all HDL subclasses was similar in both groups, AVS and CAD (Table 2). However, the content of triglycerides was 25%, 50%, and 93% higher in small HDL 3a, HDL 3b, and HDL 3c, respectively. In addition, the phospholipid concentrations of large HDL, i.e., HDL 2b and HDL 2a, were higher in the CAD group than AVS patients (Table 2).

**Figure 4:**
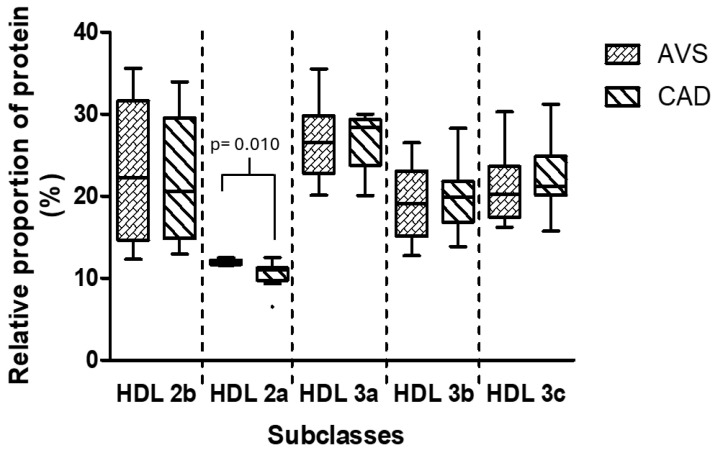
Relative proportion of HDL subclasses as a function of the content of total protein. AVS: aortic valve stenosis; CAD: coronary artery disease; HDL: high-density lipoproteins. Data are shown as the median and interquartile range (box and whisker plot). Mann–Whitney *U*-test.

Concerning HDL size distribution, the CAD group had a slightly but significantly lower relative proportion of HDL 2a than AVS patients (Figure 4).

## 4. Discussion

In this study, we demonstrated that stimulating cardiac adipocytes with HDL isolated from CAD or AVS patients induced an increase in *OPN* gene expression, whose proteinic product has been related to calcifying processes [25,26]. In addition, only HDL from AVS patients gave rise to a lower expression of *BMP4*, another pro-calcifying protein, and an increase in *LEP* gene expression. Also, there were slight but statistically significant differences in HDL structure; notably, CAD patients had a higher plasma concentration of triglycerides and phospholipids of small and large HDL particles, respectively. Finally, we demonstrated that in vitro differentiated cardiac adipocytes internalize lipids, particularly cholesterol, transported by rHDL.

We have previously demonstrated that epicardial adipose tissue from CAD and AVS patients expressed genes related to the calcification of tissues [14]. In that study, there was a statistical association between the components and proportions of HDL subclasses with gene expression [14]. In the same way, other studies have also suggested associations between mRNA expression and HDL plasma levels [27,28]. Such pieces of evidence brought about the hypothesis that HDL may directly regulate the gene expression of epicardial adipose tissue. With this idea in mind, we first developed a model of in vitro differentiated adipocytes; we were interested in cells derived from cardiac adipose tissue since it is known that the different visceral adipose tissue depots do not share the same embryonic origin [29,30], and consequently, their metabolic behavior is also different [31]. Therefore, we used the stromal fraction of rabbit cardiac adipose tissue to establish our cellular model. To assure homogeneous cell responses to HDL stimulation in all the stages of this study, we used the same primary culture of stromal cells for all the experiments. In addition, the low dispersion of *PER* gene expression was a marker of a similar rate of differentiation from stromal cells to adipocytes. After differentiation induction, the cells in the culture fulfilled the characteristics of adipocytes, i.e., cytoplasmatic lipid droplets and mRNA expression of *LEP*, *UCP*, and *PER*. With this cellular model, we explored the potential capacity of HDL to modify the mRNA levels of the genes of interest.

As previously mentioned, we found a statistical association between the composition and relative proportion of HDL subclasses with the expression of genes related to atheroma calcification expressed in epicardial adipose tissue [14]. Therefore, we isolated HDL from 21 AVS or CAD patients, both calcifying cardiovascular disorders, to determine the potential effect of HDL on regulating the expression of genes related to calcification and specific genes of adipose tissue. In addition to SBP, there were no statistical differences in the clinical parameters between both groups of patients, in agreement with the similitude linking the two diseases; it has been demonstrated that AVS shares risk factors with CAD, such as alterations in the circulant lipid concentrations and the infiltration of LDL in the valve [14,32]. Importantly, a previous study [33] showed significant differences between CAD and AVS patients, including cholesterol, glucose, and HDL-C, but such differences may be attributed to age and BMI since the AVS patients were younger, whereas the CAD patients’ BMIs were higher than that of the AVS group. In contrast, in the present study, the groups were more homogenous; particularly, age and BMI did not differ between the groups.

Then, our cell model of adipocytes was incubated with HDL isolated from AVS or CAD patients. The final concentration of cholesterol from the HDL used in these experiments was 25 mg/dL, similar to the physiological concentration in rabbits [34,35]. Under these conditions, the HDL from the AVS patients promoted the gene expression of *OPN* and *LEP* and decreased the *BMP4* gene expression. In contrast, the HDL from the CAD group promoted only the expression of *OPN* compared to control cells. These results demonstrate the contribution of HDL in regulating mRNA levels in adipocytes. In agreement, previous reports have demonstrated that HDL may modulate the mRNA of cytokines in cultured macrophages [36] and the mRNA of genes related to fatty acid metabolism [37], supporting the idea that HDL have an intrinsic property of affecting gene expression. Importantly, not all the analyzed mRNAs were modified by the presence of HDL in the culture media; *UCP* and *PER*, two characteristic genes of adipocytes, were not significantly modified by the HDL. This result implies a selective effect of HDL on genes related to the secretory activity of adipocytes but not to the browning of adipose tissue. Also, the HDL from the CAD and AVS patients did not have the same impact on mRNA expression. Taken together, these observations suggest cellular mechanisms that target specific genes in an HDL-composition-dependent manner.

**Figure 5:**
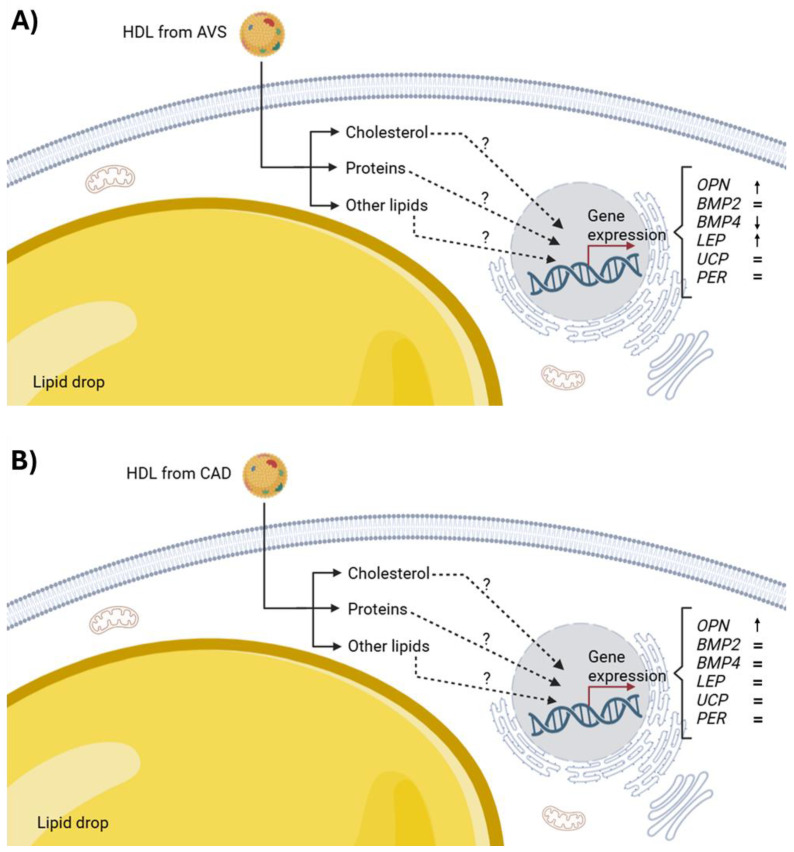
Effect of HDL isolated from AVS patients (**A**) or CAD patients (**B**) on the expression of the genes related to tissue calcification (*OPN, BMP2,* and *BMP4*) and characteristic genes of the adipocyte phenotype (*LEP, UCP,* and *PER*). HDL are internalized into adipocytes (solid lines), and their different components affect gene expression by unknown mechanisms (dotted lines). Differences in the composition and/or structure of the HDL from the AVS or CAD patients are likely the origin of the differential effects on gene expression.

The mechanisms involved in the modulation of gene expression by HDL are still unknown. From this perspective, we propose that HDL are bidirectional vectors that deliver different lipids and even proteins to cells [9,15]. Previous reports demonstrated that HDL protein and HDL sphingomyelin internalization to the HMEC-1 cells are partially dependent on scavenger receptor class B type I (SR-BI), whereas HDL-C internalization is SR-BI independent [15]. The mechanisms of HDL internalization are likely similar in adipocytes, but this similitude remains to be demonstrated. Such delivery of HDL lipids to endothelial cells in culture was fundamental in decreasing ICAM-1 and activating the eNOS [15]; therefore, HDL may also affect other cell responses, i.e., regulating gene expression. By this means, lipids provided by HDL maintain the integrity of the lipid rafts [9,38,39,40] and the adequate fluidity and functionality of both the cytoplasmic and the internal membranes [9,38,39,40,41,42], including the endoplasmic reticulum [41,42,43,44,45]. Consequently, HDL may contribute to modulating the expression of certain mRNAs. This hypothesis implies that the lipid cargo of HDL will have an important impact on adipocytes after the internalization of these lipoproteins. Considering that HDL must be internalized to deliver their lipid cargo [15], as demonstrated in endothelial cells, macrophages, and cancer cells [15,46], it was fundamental to demonstrate that adipocytes also internalize HDL lipids. Using rHDL containing fluorescent cholesterol, we demonstrated that cardiac adipocytes internalize lipids from these lipoproteins into their cytoplasm. In other words, cholesterol and the influx of other lipids seem to be a common phenomenon in most cells. Interestingly, the cholesterol from HDL was not incorporated into the lipid drop. In addition, these results suggest the usefulness of rHDL as lipid delivery nanoparticles to adipose tissue. Figure 5 illustrates the main findings of the effects of the HDL isolated from the CAD and AVS patients on gene expression.

Since there was an influx into adipocytes of rHDL cholesterol and HDL protein, other lipids and proteins could also be internalized and affect gene expression. Therefore, the structure and composition of HDL are potential factors that might help to explain the different gene expression in adipocytes. In this context, we characterized the HDL structure isolated from CAD and AVS patients. It is known that HDL are structurally modified in several pathologies; particularly, a high triglyceride content has been associated with HDL dysfunction [1,47,48]. We also observed an increased content of triglycerides in the small HDL from the CAD patients in comparison with the AVS group, suggesting that an augmented influx of this lipid into the adipocytes via HDL may affect gene expression, a hypothesis that merits exploration in further studies. Other structural differences were the reduction in the HDL 2a protein and increased levels of phospholipids in the large HDL2b and HDL2a subclasses in the CAD patients. Whether such differences in HDL structure have a real impact on adipocyte gene regulation remains to be elucidated.

The structural differences between the HDL isolated from the CAD patients with respect to the HDL from the AVS group could impact the regulation of gene expression observed in this study. In addition, it should be emphasized that HDL are complex aggregates of molecules, including a great number of proteins in addition to apolipoproteins, including inflammation factors, acute-phase proteins, and coagulation factors, among others [1,49,50,51,52,53]. Since HDL become internalized into cells [15], the abnormal protein cargo may also contribute to the observed differences in mRNA levels in adipocytes stimulated with the HDL from the CAD and AVS patients. Even if we demonstrated the rHDL uptake by adipocytes and some structural differences between the HDL isolated from the CAD and AVS patients, we recognize as a limitation of the study that we did not demonstrate a cause–effect relationship between HDL structure and gene regulation. More studies are needed to analyze the proteomics and lipidomics of HDL in both physiopathological conditions and whether the HDL structure may predict the gene regulation in adipocytes.

On the other hand, the genes that were modulated in cardiac adipocytes by the HDL tested in this study may have important implications. Myasoedova et al. demonstrated a positive association between plasma leptin levels and the development of aortic valve stenosis [54]. In addition, a higher expression of leptin could induce the transformation of smooth muscle cells from the aorta into osteogenic cells [55], contributing to the secretion of specific calcification factors in the aortic valve [56]. Congruently, the HDL isolated from the AVS patients induced a higher expression of *LEP* in the cardiac adipose cells, suggesting a potential contribution of these lipoproteins to the calcium deposit in the aortic valve that merits exploration in future studies.

OPN is a versatile protein that is poorly understood [26] that could maintain a chronic inflammatory microenvironment. In this context, our results suggest that the HDL from the CAD or AVS patients may contribute to the calcification of either the atheroma or the aortic valve by enhancing the gene expression of *OPN*.

Finally, BMP-4 not only is a pro-calcifying protein but also maintains the homeostasis of adipose tissue by regulating adipogenesis [57], inducing the browning of white adipose tissue [58], and reducing the production of proinflammatory adipocytokines [57]. Therefore, we cannot discard the possibility that dysfunctional HDL can contribute to EAT hypertrophy by downregulating the gene expression of *BMP4*. Future studies are necessary to demonstrate this hypothesis.

Our findings may have some clinical implications; we demonstrated a real cause–effect relationship between HDL and gene expression, as was suggested by the statistical associations previously reported in CAD and AVS patients [14]. Therefore, based on the present results, the triglyceride content in HDL may represent a future target to limit the inflammation and calcium burden in coronary arteries. Concerning the aortic valve, HDL may enhance calcium deposition in the valve through other components not explored in this work, such as sphingomyelins [15,59]. Consequently, models of cells characteristic of the aortic valve, such as fibroblasts, smooth muscle cells, and myofibroblasts [60], are needed to establish whether HDL lipids, beyond cholesterol, are a potential therapeutic target for aortic valve calcification prevention.

We recognize the limitations of our study, including the small number of patients enrolled in the study. Also, the extrapolation from our cell model to physiopathology in vivo is not possible since the metabolic and genetic similitudes between cardiac stromal cells from rabbit and human cardiac adipose tissue have not been explored. Therefore, our results should be interpreted carefully; we only provide evidence that HDL, at physiological concentrations, can modulate the mRNA of certain genes related to tissue calcification, particularly in adipocytes derived from cardiac adipose tissue. In addition, rHDL with different lipid compositions and their impact on the gene expression in cardiac adipose tissue is another area of opportunity that should be considered in future studies.

In summary, we developed a model of cardiac adipocytes to demonstrate that HDL isolated from AVS or CAD patients differentially modulated the expression of genes related to calcification and to the phenotypic characteristics of these cells. One potential mechanism explaining this HDL function is the lipid delivery to the cells, as demonstrated with rHDL containing cholesterol labeled with a fluorescent dye. Therefore, the high concentration of triglycerides of small HDL particles and phospholipids in the large HDL from the CAD patients compared to that isolated from the AVS group may be associated with the different mRNA expression in cardiac adipocytes. However, other components of HDL may be also involved, and further research is guaranteed in this field.

## 5. Conclusions

HDL isolated from AVS or CAD patients differentially modulated the expression of genes related to calcification and the phenotypic characteristics of cardiac adipocytes. Such a differential effect occurred concomitantly with a high concentration of small HDL triglycerides and a higher concentration of phospholipids in the large HDL from the CAD patients than the HDL from the AVS group. We also demonstrated for the first time that rHDL lipids are internalized to adipocytes. Taken together, these results suggest that the potential mechanism explaining the role of HDL on gene regulation is the delivery of lipids to the cardiac adipocytes.

## Figures and Tables

**Table 1:** Patients’ clinical characteristics.

Parameter	AVS (*n* = 10)	CAD (*n* = 11)
Age (years)	59 (46–69)	67 (60–71)
Gender (M)	8 (80%)	11 (100%)
BMI (kg/m^2^)	25.8 (23.0–29.3)	24.7 (24.0–26.5)
SBP (mm Hg)	115.0 (105.3–120.0)	128.5 (117.5–142.5) *
DBP (mm Hg)	67.5 (60.0–73.3)	77.0 (67.5–83.3)
Ca^2+^ (mg/dL)	9.2 (8.6–9.5)	9.4 (8.7–9.6)
Alkaline phosphatase (U/L)	101.7 (71.8–129.6)	74.8 (67.5–98.9)
Glucose (mg/dL)	84.2 (73.6–147.8)	91.2 (72.3–100.3)
Cholesterol (mg/dL)	119.6 (88.7–152.8)	119.0 (99.4–132.0)
TG (mg/dL)	97.4 (72.7–120.4)	119.2 (89.2–142.7)
LDL-C	73.0 (41.0–92.8)	61.7 (39.3–73.6)
HDL-C (mg/dL)	33.9 (26.0–34.8)	30.5 (24.3–37.5)
HDL-TG (mg/dL)	17.0 (11.7–21.1)	19.6 (17.2–22.1)
HDL-PH (mg/dL)	54.8 (48.5–65.0)	68.3 (59.9–74-8)
Statins treatment	5 (50%)	8 (73%)
CAC (AU)	0 (0–0)	412.3 (60.4–1484)

AVS: aortic valve stenosis; CAD: coronary artery disease; BMI: body mass index; SBP: systolic blood pressure; DBP: diastolic blood pressure; TG: triglycerides; LDL: low-density lipoproteins; HDL: high-density lipoproteins; PH: phospholipids; CAC: coronary artery calcium; AU: Agatston units. Data are expressed as median (interquartile range) and n (percentage). Mann–Whitney *U*-test. * *p* < 0.05.

**Table 2:** Lipid composition of HDL subclasses.

Lipid (mg/dL)	Group	HDL 2b	HDL 2a	HDL 3a	HDL 3b	HDL 3c
Cholesterol	AVS	11.8 (11.5–16.5)	4.5 (3.8–5.7)	6.9 (5.7–9.1)	2.8 (2.3–4.5)	2.5 (1.8–3.6)
	CAD	12.7 (10.2–17.3)	4.6 (3.8–6.2)	6.9 (5.3–9.3)	3.1 (2.3–3.5)	2.8 (2.3–3.7)
Triglycerides	AVS	7.4 (3.6–9.5)	2.8 (1.4–3.7)	4.0 (2.9–4.8)	1.6 (1.30–1.8)	1.4 (1.0–1.6)
	CAD	7.5 (4.4–10.3)	2.8 (2.4–3.9)	5.0 (4.4–5.7) *	2.4 (1.6–3.3) *	2.7 (1.5–3.0) *
Phospholipids	AVS	22.7 (18.4–26.0)	8.3 (7.5–9.5)	13.8 (10.5–15.4)	6.6 (3.1–7.2)	5.9 (2.7–7.1)
	CAD	28.7 (25.0–29.6) *	10.8 (8.7–11.6) *	15.6 (13.5–16.15)	6.4 (5.5–7.6)	6.1 (4.2–9.1)

AVS: aortic valve stenosis; CAD: coronary artery disease; HDL: high-density lipoproteins. Data are expressed as median (interquartile range). Mann–Whitney *U*-test. * *p* < 0.05.

## Data Availability

The raw data supporting the conclusions of this article will be made available by the authors on request.

## Supplementary Materials

The following supporting information can be downloaded at https://www.mdpi.com/article/10.3390/cells14030205/s1, Table S1: Primers used for gene expression.
