# Supplementary material for: High-Density Lipoproteins from Coronary Artery Disease and Aortic Valve Stenosis Patients Differentially Regulate Gene Expression in a Model of Cardiac Adipocytes
Source: Cells. 2025 Jan 30;14(3):205. doi: 10.3390/cells14030205 (PMC11817163; doi:10.3390/cells14030205)
Supplement: Supplementary file 1 [file cells-14-00205-s001.zip › cells-3368718-supplementary.pdf]

Supplementary table 1. Primers used to gene expression

Gene	Forward	Reverse	Tm (°C)
OPN	5´AGACCTCCCGAGTAAGTCC 3´	5′GTGGTCATCGTCCTCATCCT 3′	58.2
BMP2	5′ AGACGACAGCGGTTTCCATC 3´	5′CGGCTCGTGTTCTGATTCAC 3′	59.4
BMP4	5′ CTCTGTCAACTCCAGCATCC 3′	5′GCACCCACACCCCTCTACTA 3′	58.2
LEP	5´GACAGAGGGTCGTCGGTTTG 3´	5´GTTCTCCAGGTCGTTGGCT 3´	61.0
UCP	5´ACGCTTCTGTCCTCTCCAGT 3´	5´GCCGTTGGTCCTTCCTTAGT 3´	59.4
PER	5´GAAGTCGGATGGTGCAGTT 3´	5´AGGGGGAGGTATTGGTCAAC 3´	59.4
GAPDH	5′TGGTGAAGGTCGGAGTGAAC3′	5′GGCGACAACATCCACTTTGC 3′	59.4
